# Inhibition of Proliferation in U937 Cells Treated by Blue Light Irradiation and Combined Blue Light Irradiation/Drug

**DOI:** 10.3390/ijms19051464

**Published:** 2018-05-15

**Authors:** Jianjian Zhuang, Junsong Liu, Xuan Gao, Hongdong Li

**Affiliations:** State Key Laboratory of Superhard Materials, Jilin University, Changchun 130012, China; zhuangjianjian1234@163.com (J.Z.); jsliu@jlu.edu.cn (J.L.); gaoxuan16@mails.jlu.edu.cn (X.G.)

**Keywords:** enhanced proliferation inhibition, U937 cells, BL irradiation, apoptosis

## Abstract

The cell viability and apoptosis of tumor U937 cells treated by blue light (BL) irradiation have been examined. BL irradiation can specially inhibit the proliferation and promote the apoptosis of U937 cells, relating to the production of reactive oxygen species (ROS) and the decline of mitochondrial membrane potential (ΔΨm). The apoptosis is further associated with varying downregulated *B-cell lymphoma-extra large* (*Bcl-X_L_*) and *B-cell lymphoma 2* (*Bcl-2*) genes, upregulated *Bcl-2-associated X* (*Bax*) gene, the activation of caspase-3 and caspase-9, and the cleavage of poly (ADP-ribose) polymerase (PARP) by the BL irradiation process. Moreover, BL irradiation induced proliferation inhibition is higher than that treated by a common chemotherapeutic drug of homoharringtonine (HHT). When we synergize BL irradiation with HHT (BL-HHT), a higher proliferation inhibition is obtained than that treated by BL irradiation or HHT alone. These results are helpful for establishing a low toxicity and high efficiency strategy of BL irradiation for clinical treatment of acute myeloid leukemia, not limited to U937 cells.

## 1. Introduction

Acute myeloid leukemia (AML) is a group of hematopoietic malignancies arising from the abnormalities of proliferation, differentiation, or survival of myeloid progenitors [1]. U937 with diffuse histiocytic lymphoma features becomes an important malignant tumor cell model [2], belong to AML-M5 subtype (by French–American–British classification) [3]. The common drugs contain cytosine carabinoside, daunorubicin, Adriamycin [4], or homoharringtonine (HHT), et al. Among them, HHT is an antitumor alkaloid isolated from the natural plants, and it has less drug toxicity than other chemotherapy drugs [5,6,7], and thus HHT is generally proposed for the treatment of AML-M5. However, the side effects, such as cardiovascular complications, tachycardia, and hypotension, might occur with increased drug dosage [8,9]. To improve leukemia treatment and decrease the side effects, the composite medicines are prepared to obtain high complete remission rate [7,10], while long time cytarabine or aclarubicin treatment are always accompanied with fatal complications [11]. Another strategy is introducing nanomaterials as a form of drug delivery that can increase the bioavailability and solubility of the drugs [12], but it is difficult to avoid toxicity [13] and aggregation [14] related to small-sized nanomaterials. Therefore, it is highly desirable to explore a new strategy to further improve therapeutic efficiency and minimize side effects in the treatment of U937 cells.

It is known that phototherapy has been widely used in tumor therapy, including photodynamic therapy (PDT) and photothermal therapy (PTT). PDT is a treatment procedure involving the reactive oxygen species (ROS) generation when the photosensitizing (PS) drug is irradiated by lasers and light emitting diodes (LEDs) at certain wavelengths corresponding to the absorption spectrum of the PS agent [15]. PTT is a therapeutic method based on the use of near-infrared (NIR) light that activates photosensible nanomaterials, resulting in localized photothermal effects [16]. In fact, without photosensitizer and photothermal treatments, the cold-light irradiation (e.g., BL related fluorescent lamp) has been performed on the clinical treatments of acne and hyperbilirubinemia for newborns [17,18]. Some tumor cells, such as human acute promyelocytic leukemia cells (HL60) [19,20,21], melanoma cells (B16) [22], B cell lymphoma cells (A20) [23], and human colon cancer cells (HCT116) [24], can be inhibited to a certain extent by BL irradiation. 

In this paper, the U937 cells are treated by BL irradiation to achieve a significant proliferation inhibition. By examining reactive oxygen species (ROS), mitochondrial membrane potential (ΔΨm), expression of mitochondrial apoptosis related genes (upregulated *Bcl-2-associated X* (*Bax*)*,* downregulated *B-cell lymphoma-extra large* (*Bcl-X_L_*), and *B-cell lymphoma 2* (*Bcl-2*)), and apoptosis-related proteins (activation of caspase-3, caspase-9, and cleavage of poly (ADP-ribose) polymerase (PARP)), the mechanisms of the inhibition and apoptosis are discussed. It is demonstrated that the treatment of BL irradiation is favorable for enhancing the inhibition of U937 cells and decreasing the side effects, and the treatment efficiency is higher than that by some common chemotherapeutic drugs.

## 2. Results and Discussion

**Figure 1:**
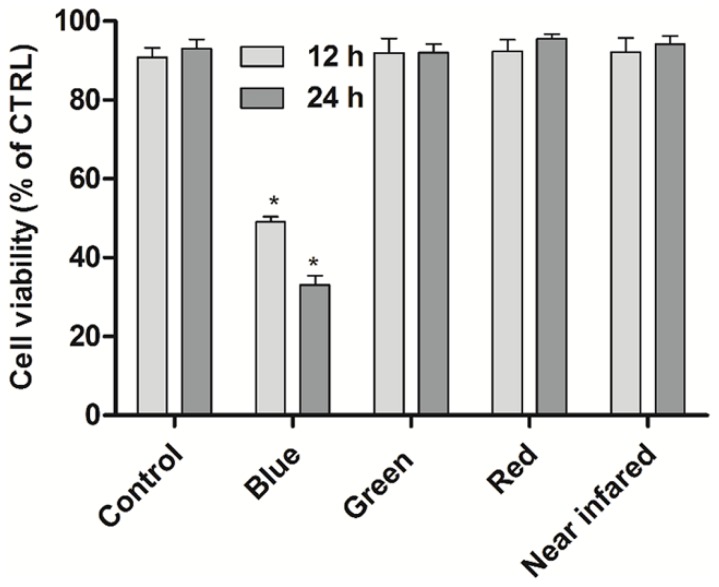
The proliferation inhibition of U937 cells, irradiated by different colored light emitting diodes (LED) arrays with wavelengths centered at 456 nm (blue), 515 nm (green), 630 nm (red), and 840 nm (near infrared) for 12 and 24 h, without incubation. The cell viabilities are evaluated using Cell Counting Kit-8 assay (CCK-8) assay for 4 h, and the absorbance values are measured at 450 nm. Data shown are the mean values ± SD of at least three independent experiments. * *p* < 0.05 vs. control group. The cells treated under dark conditions is set to control group.

Firstly, to examine the specificity of BL irradiation, the LED arrays with different wavelengths are used to examine the proliferation inhibition of U937 cells. In Figure 1, significant proliferation inhibition of U937 cells is achieved under BL irradiation (with wavelength centered at 456 nm [20]), which is higher than other colored LEDs at various wavelengths centered at 515, 630, and 840 nm.

After BL irradiation was performed on U937 cells for 2, 4, and 8 h, the proliferation inhibition of the treated cells incubated to 0, 12, and 24 h are examined. As shown in Figure S1, compared to the control, the proliferation inhibition rates of U937 cells increase after BL irradiation for 2, 4, and 8 h (14.4%, 48.7%, and 72.7%, respectively). This means that the inhibition rate increases with increasing irradiation time. When the cells are incubated for 12 and 24 h without continuous BL irradiation, the proliferation inhibition rates keep increasing. These results demonstrate that the proliferation inhibition of U937 cells has been triggered by BL irradiation for 2 h, and consequently, 2 h irradiation treatment of BL is performed in the following discussions.

The treatment of drug HHT is introduced to compare with BL irradiation on U937 cells. In Figure S2, although HHT can induce the proliferation inhibition of U937 cells, the rates are less than 50% at the concentrations of 0.05 to 0.1 μg/mL, which are lower than those treated by BL irradiation.

**Figure 2:**
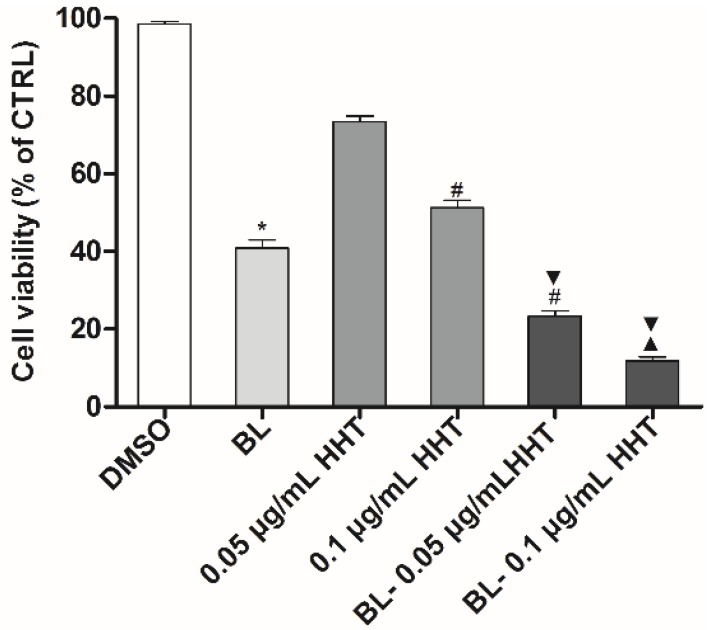
The cell viability of U937 cells treated under various conditions with 0.1% dimethyl sulfoxide (DMSO) medium alone (control), blue light (BL), homoharringtonine (HHT), and BL-HHT. The BL treated group is incubated for 24 h after 2 h irradiation, the HHT group is incubated for 24 h without irradiation, and the BL-HHT group is incubated for 24 h after 2 h irradiation. After incubated for 24 h, the cell viabilities are evaluated using CCK-8 assay for 4 h, and the absorbance values are measured at 450 nm. Values shown are the means ± SD (*n* = 3). * *p* < 0.05 vs. 0.1% DMSO, # *p* < 0.05 vs. 0.05 μg/mL HHT, ▼ *p* < 0.05 vs. BL, ▲ *p* < 0.05 vs. 0.1 μg/mL HHT.

It is expected that combined BL irradiation with HHT could further enhance the treatment effect of U937 cells. Indeed, Figure 2 shows that the proliferation inhibition ratios treated by BL irradiation and 0.05 (0.1) μg/mL HHT can be as high as 76.7% (88.1%), which are higher than those of cells treated by HHT or BL irradiation alone.

**Figure 3:**
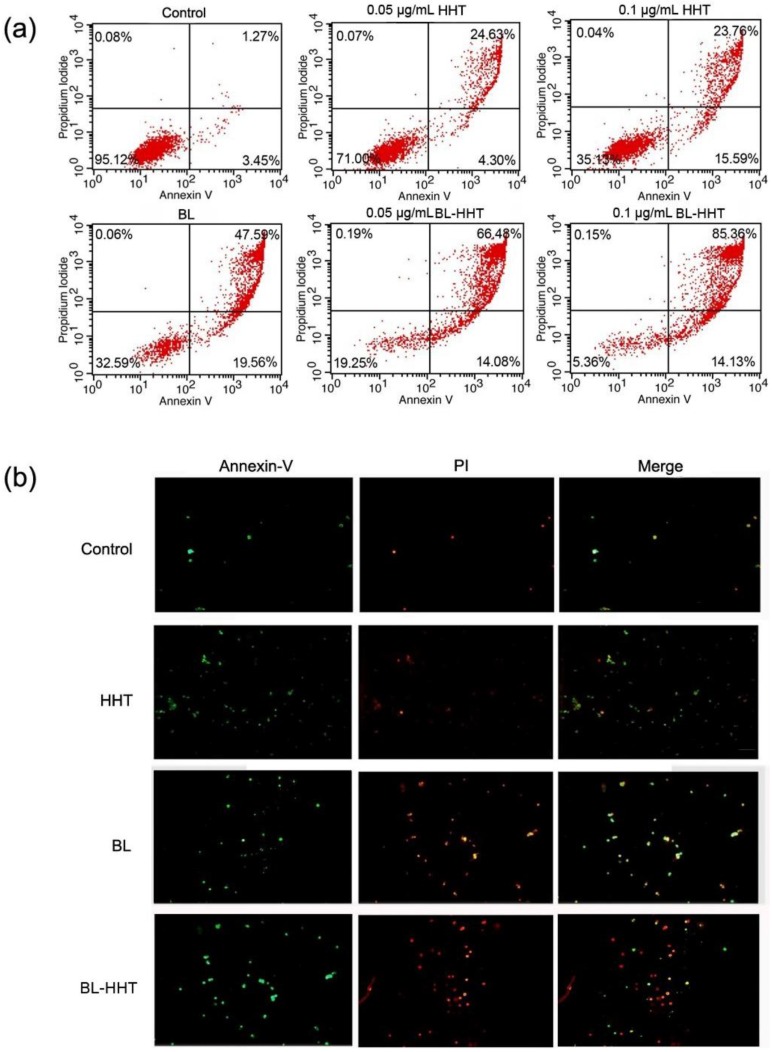
Assessing the cell apoptosis ratios of U937 cells treated under various conditions with 0.1% DMSO medium alone (control), BL, HHT, and BL-HHT treatments. The BL treated group is incubated to 24 h after 2 h irradiation, the HHT group is incubated for 24 h without irradiation, and the BL-HHT group is incubated to 24 h after 2 h irradiation. After incubation for 24 h, the apoptosis ratios are detected by flow cytometry. The identification of the fluorochromes are measured and analyzed by FACScan flow cytometry for Annexin V–FITC and PI. (**a**) The apoptosis ratios are calculated by the Cell Quest software (Becton Dickinson). FACS analysis indicated that the total apoptosis ratios include apparent early apoptosis (lower right (LR) quadrant) and late apoptosis (upper right (UR)); (**b**) Annexin V–FITC and PI staining of U937 cells detected by fluorescence light microscopy (magnification 40×) after treatment with various groups. The green and red fluorescence represent the early apoptosis cells and late apoptosis, respectively.

The apoptosis of U937 cells treated by BL irradiation and HHT are measured by annexin V- fluorescein isothiocyanate (Annexin V-FITC) and propidium iodide (PI) staining method to study the mechanism of the varying proliferation inhibition. In Figure 3, 67.15% apoptosis ratio is realized by BL irradiation treatment, which is higher than that by HHT (0.05 μg/mL 28.93%; 0.1 μg/mL 39.35%). When combining BL irradiation (2 h) and HHT treatment for 24 h, the apoptosis of U937 cells further enhances (80.56% for BL-0.05 μg/mL HHT; 99.49% for BL-0.1 μg/mL HHT) with respect to that treated by BL irradiation or HHT alone. 

**Figure 4:**
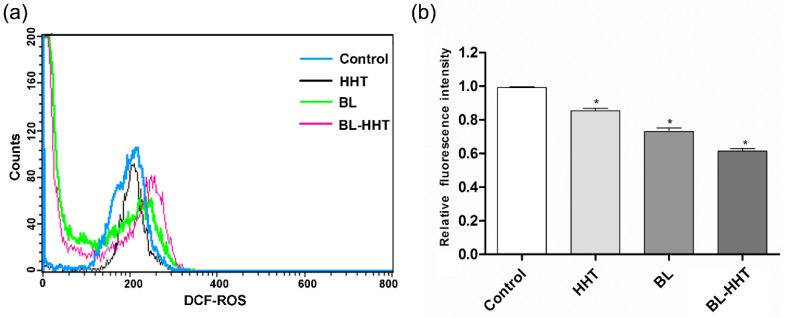
(**a**) The level of reactive oxygen species (ROS) content in U937 cells, detected by fluorescent probe of H_2_DCFDA; (**b**) The dissipation of ΔΨm, detected via 5,5’,6,6’-tetrachloro-1,1’,3,3’-tetraethylbenzimidazolcarbocyanine iodide (JC-1) staining and analyzed by FACScan flow cytometry. The cells were treated with 0.1% DMSO medium alone (control), BL, HHT, and BL-HHT. The BL irradiation group is incubated for 24 h after 2 h irradiation, the HHT group is incubated for 24 h without irradiation, and the BL-HHT group is incubated for 24 h after 2 h irradiation. After the treatment, the cells are incubated with 2',7'-dichlorodihydrofluorescein diacetate (H_2_DCFDA) and JC-1 for 30 min. The contents of ROS are calculated by measuring the fluorescence intensity of dichlorodihydrofluorescein (DCF). The decreasing relative proportion of red and green fluorescence represents the loss of ΔΨm. Values shown are the means ± SD (*n* = 3). * *p* < 0.05 vs. control group.

Figure 4a shows the production of ROS, and Figure 4b shows the decline of ΔΨm in U937 cells treated by BL irradiation. The porphyrin contained in enzymes from mitochondria are proposed as acceptors for BL irradiation [25,26], which would produce a large amount of ROS and lead to the final apoptosis. The above results suggest that the apoptosis caused by BL irradiation are related to both the ROS and mitochondrial membrane permeabilization (MMP). For HHT, the content of ROS is nearly the same as that for the control group, indicating that the apoptosis response by HHT is ROS independent [27]. The decline of ΔΨm treated by HHT is presented, meaning that the cell apoptosis does not involve ROS, but mainly relies on the decline of ΔΨm.

**Figure 5:**
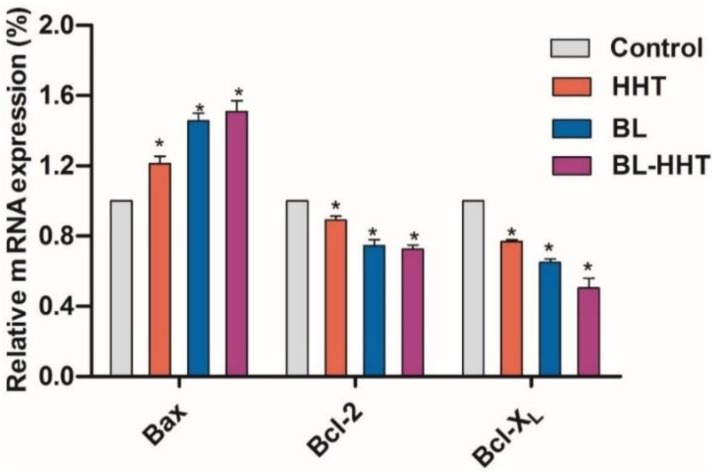
The expression level of *Bax, Bcl-2, Bcl-X_L_* mRNA in U937 cells determined by using Real-time fluorescent quantitative Polymerase Chain Reaction (qRT-PCR). The cells were treated with 0.1% DMSO medium alone (control), BL, HHT, and BL-HHT. The BL treated group is incubated for 24 h after 2 h irradiation, the HHT group is incubated for 24 h without irradiation, and the BL-HHT group is incubated to 24 h after 2 h irradiation. The qRT-PCR is performed using the ABI Prism 7500 Sequence Detection System. Values shown are the means ± SD (*n* = 3). * *p* < 0.05 vs. control group.

Generally, MMP plays an important role in keeping mitochondrial stable, which might determine cell apoptosis [28,29]. In most tumor cells, the mitochondrial membrane potential is controlled by anti-apoptotic genes (e.g., *Bcl-2, Bcl-X_L_*) and pro-apoptotic genes (i.e., *Bax*) [30]. The expression of these genes is detected by qRT-PCR to explore the molecular mechanism of proliferation inhibition of U937 cells induced by BL irradiation. In Figure 5, the expression of *Bcl-X_L_* and *Bcl-2* after BL irradiation decreases, corresponding to those of the control and treated by HHT, while the expression of *Bax* has an opposite relationship between the two cases. This implies that the apoptosis of U937 cells triggered by BL irradiation is mainly attributed to the increasing expression of pro-apoptotic genes (i.e., *Bax*), and at the same time, to the suppressing expression of anti-apoptotic genes (e.g., *Bcl-2, Bcl-X_L_*). Treated by drug HHT, the expressions of *Bcl-X_L_* and *Bcl-2* decrease, while the *Bax* increases, which is similar to previous reports [31,32], and when treated by BL irradiation. Note that the ratios of *Bcl-2/Bax* (0.73) and *Bcl-X_L_/Bax* (0.63) for HHT treatment are higher than that for BL irradiation treatment (*Bcl-2/Bax*, 0.53; *Bcl-X_L_/Bax*, 0.46), suggesting that the BL irradiation is a more efficient treatment for the proliferation inhibition of U937 leukemia cells, rather than HHT. Combining BL irradiation and HHT for the treatment of U937 cells, the enhanced (decreased) expression of *Bax* (*Bcl-2, Bcl-X_L_*) gene increases the inhibition rate of U937 cells with respect to those treated by either BL irradiation or HHT. Importantly, it is found that for the anti-apoptotic *Bcl-X_L_* genes, expression has been significantly suppressed after combined BL-HHT treatment. This suggests that the synergistic effect of BL irradiation and HHT could enhance the proliferation inhibition of U937 cells (in Figure 2) mainly by suppressing the anti-apoptotic *Bcl-X_L_* genes. 

**Figure 6:**
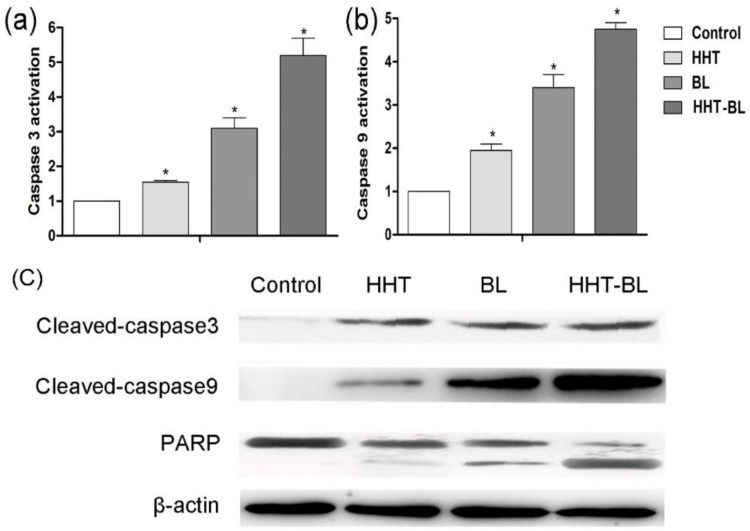
Variations of the apoptosis-related proteins in U937 cells after treatments by BL, HHT, and BL-HHT. The BL-treated group is incubated for 24 h after 2 h irradiation, the HHT group is incubated for 24 h without irradiation, and the BL-0.05 μg/mL HHT group is incubated for 24 h after 2 h irradiation. (**a**) The activity of caspase-3, (**b**) caspase-9 are measured using protein activity assay; (**c**) The cleaved caspase-9, cleaved caspase-3, and cleaved-PARP are determined via immunoblotting assay. β-Actin is used as a loading control. Bars of means ± SD are obtained from three independent cell culture experiments. * *p* < 0.05 vs. control group.

We further investigate the possible role of BL irradiation in controlling the apoptosis of U937 cells through the activation of caspase proteins. As shown in Figure 6a, b, the activities of caspase-3 and caspase-9 proteins treated by BL irradiation are 2.0 and 1.7 times higher than those treated by HHT, respectively. Furthermore, in Figure 6c, the cleaved caspase-3 (caspase-9) and PARP treated by BL irradiation are 3.1 (1.1) and 2.0 times higher than that treated by HHT. These results suggest that the BL irradiation enhances proliferation inhibition of U937 cells by improving the activation of caspase-3 and caspase-9, and cleavage of PARP, which is more efficient than in those treated by the conventional HHT drug [33,34]. Moreover, it is expected that the synergistic BL-HHT can further enhance the inhibition of U937 cells by improving the activities of caspase-3 and caspase-9 proteins, and cleavage of PARP.

Finally, to explore possible side effects of BL irradiation on the normal cells, normal blood cells of PBMC are selected to be tested. The cells have been stimulated by PMA/ionomycin and CD3/CD28 antibody to promote the proliferation of T-cells in the PBMC, acting as a positive control. As shown in Figure S3, after BL irradiated for 2 h, these PBMC have the similar viability ratio as the control group, and we further conclude that the BL irradiation achieves relatively low inhibition rates, as low as ~10% even following long incubation times (12, 24, and 48 h), in contrast to the inhibition of U937 cells, having higher inhibition rates up to ~75% (Figure S1). It is thus confirmed that there is no evident proliferation inhibition of PBMC, which is consistent with previous reports [19]. It is demonstrated that the BL irradiation does not strongly influence the proliferation of normal blood cells, by contrast, the U937 cells are significantly inhibited by BL irradiation treatment, as well as by drugs (e.g., by HHT). The BL irradiation thus becomes an advantageous therapeutic method, with fewer side effects in comparison with the drugs. It is worth pointing out that although the tumors in the body might not be easily irradiated by BL (penetration limits of BL in biological tissue), the extracorporeal circulation therapy by BL irradiation is possible [19]. In fact, our BL treatment research, performed on leukemia HL60 cells both in vitro and in vivo, revealed a good inhibition process [20,21]. We thus propose a new concept of killing tumor cells by BL in extracorporeal circulation could be practical in clinical treatment.

## 3. Materials and Methods

### 3.1. Materials

HHT was from Yuanye Technologies Inc. (Shanghai, China). Dimethyl sulfoxide (DMSO) solvent was used to prepare the HHT, and the stock concentration of HHT was 1 mg/mL. Roswell Park Memorial Institute (RPMI) 1640 medium was from Gibco (Buffalo, NY, USA). Fetal bovine serum (FBS) was from Tianhang Biotechnology Co., Ltd. (Zhejiang, China). Cell Counting Kit-8 assay (CCK-8), Annexin-FITC Apotosis Kit, Caspase-3 Activity Assay Kit, and BCA Assay Kit were from BestBio Biotechnology Co., Ltd. (Shanghai, China). Cellular Reactive Oxygen Species Detection Assay Kit was from Beyotime Biotechnology Inc. (Jiangsu, China). Mitochondrial Potential Detection Kit and Caspase-9 Fluorescence Metric Assay Activity Kit (KGA402F-KGA404F) were from KeyGen Biotechnology Co., Ltd. (Jiangsu, China). RNA Isolation Kit (Takara code No. 9767), Reverse Transcription Kit (Takara code No. RR047A), and SYBR Premix Ex Taq™ Kit (Code No. RR820A) were from Takara Biotechnology Co., Ltd. (Dalian, China). Polyclonal rabbit anti-human cleaved caspase-3, cleaved caspase-9, PARP antibody, secondary HRP-conjugated anti-rabbit antibody were from Bioss Technologies Inc. (Beijing, China). Ultrapure water was used in all experiments.

### 3.2. Cell Line

U937 cell line was from Keygen BioTech Co., Ltd. (Jiangsu, China). The U937 cells were cultivated in suspension.

### 3.3. Peripheral Blood Mononuclear Cells (PBMC)

Human blood samples were obtained from the School of Translational Medicine, Jilin University. Concentrated white blood cells were extracted from blood, diluted three times with physiological saline, and 15 mL of diluted concentrated white blood cells was added along the wall of a 50 mL tube which contained 15 mL lymphocyte separation solution, the solution was centrifugated for 30 min at 3000 rpm, then the solution was divided into four layers from top to bottom, the circular milk white mononuclear cells located in the second layer, the isolated cells were washed with PBS at 1800 rpm for 10 min for two times, and then cultured in complete RPMI1640 medium supplemented with 100 U/mL penicillin and 100 μg/mL streptomycin and 10% FBS, at 37 °C in a 5% CO_2_ humidified atmosphere.

### 3.4. Equipments

ELX 808 microplate reader was from Bio-Tek Instruments, Inc. (Winooski, VT, USA), FACScan flow cytometry was from Becton, Dickinson and Company (Franklin Lakes, NJ, USA) and ABI Prism 7500 Sequence Detection System was from Applied Biosystems Inc. (FosterCity, CA, USA) were used.

### 3.5. Cell Culture, Drug, and LED Irradiation Treatment Conditions

The U937 cells were cultured in complete RPMI1640 medium, at 37 °C in a 5% CO_2_ humidified atmosphere. The LED light reaction chamber consisted of a black shading box with 36 commercial LED arrays which was pasted on the upside. The power was supplied by a 12 V battery. The emission spectra were tested by USB4000 spectroscopy system was from Ocean Optics Inc. (Dunedin, FL, USA), and the radiation power density was 0.25 mW/cm^2^. To explore the specific proliferation inhibitory effect of U937 cells under LED irradiation, the different colored LED arrays were chosen with wavelengths centered at 456 nm (blue), 515 nm (green), 630 nm (red), and 840 nm (near infrared). After irradiated for 12 h and 24 h without incubation, the cell viabilities were then directly evaluated. 

To determine the optimum BL irradiation time, three groups of cells were irradiated with BL for 2, 4, and 8 h, respectively, then the irradiation was stopped, and next, the as-irradiated cells were incubated under the same conditions for 0, 12, and 24 h, for the comparison (Figure S1). It is found that for the U937 cells, after 2 h BL irradiation and flowing incubation to 24 h, the nearly same inhibition effect is presented for the cases of BL irradiation for 4 or 8 h (flowing incubation to 24 h). Thus, in the following experiments and discussion, the 2 h irradiation process was performed on the tumor U937 cells.

To explore the specific proliferation inhibitory effect of BL irradiation, the isolated peripheral blood lymphocyte cells from the blood samples were irradiated by BL for 2 h, and then the as-irradiated cells were incubated for 12, 24, and 48 h. The proliferation inhibition of U937 cells dependent on HHT concentration was discussed in Figure S2, where the cells were treated by HHT with different concentrations (0.02, 0.04, 0.05, 0.06, 0.08, and 0.1 μg/mL) for 24 h. After various treatments, the cells were incubated with 10 μL CCK-8 solution for 4 h, and the optical density at 450 nm was measured using a microplate reader. The optimum irradiation time, the specific proliferation inhibitory effect and the optimum HHT concentration in U937 cells were studied by the CCK-8 assay. The U937 cells were seeded at a density of 1.5 × 10^4^ cells/well in 96-well plates.

### 3.6. Growth Inhibition Assay

Cell growth inhibition was evaluated using the CCK-8 assay. Cells at a density of 1 × 10^4^ cells/well were seeded in 96-well plates at 37 °C in a 5% CO_2_ humidified atmosphere. The BL-treated group was incubated to 24 h after 2 h irradiation, the 0.05 μg/mL (0.1 μg/mL) HHT group were incubated for 24 h without irradiation, and the BL-0.050 μg/mL (0.1 μg/mL) HHT group was incubated for 24 h after 2 h irradiation. During all the incubation processes, the BL irradiation was removed. The U937 cells cultured in complete RPMI1640 medium with an equal amount of 0.1% DMSO only was set as the control group. After incubation for 24 h, the cell viabilities are evaluated using CCK-8 assay for 4 h at 37 °C, and the absorbance was measured at 450 nm using ELX 808 microplate reader. 

### 3.7. Apoptosis Analysis

The U937 cells apoptosis was examined using Annexin V–FITC Apoptosis Detection Kit. The U937 cells were seeded at a density of 5 × 10^5^ cells/well in 6-well plates. The cells were treated under various conditions. After treatment, the cells were collected and washed with cold PBS and incubated in 500 µL Annexin V binding buffer containing 2 µL FITC–Annexin V for 15 min, and then resuspended with propidium iodide in dark for 5 min at 4 °C. The fluorescence intensity was measured using FACScan flow cytometry for Annexin V–FITC and PI. The apoptosis ratios were analyzed using the Cell Quest software (version 3.3) (San Diego, CA, USA).

### 3.8. Membrane Potential Assessment

The membrane potential (ΔΨm) was analyzed using the JC-1 Mitochondrial Potential Detection Kit. The U937 cells were seeded at a density of 5 × 10^5^ cells/well in 6-well plates, the cells were treated under various conditions. After treatment, the cells were washed twice with cold PBS and stained with JC-1 for 30 min at 37 °C in the dark. The cell suspension was filtered through 400-mesh nylon, and then the fluorescent intensity of JC-1 was measured by FACScan flow cytometry.

### 3.9. ROS Determination

The cellular ROS was quantified using cellular ROS Detection Assay Kit. The U937 cells were seeded in 50 mm plates at a density of 1 × 10^6^ cells/well. The cells were treated under various conditions. After treatment, the cells were incubated with 10 mM H_2_DCFDA (1:1000 dilution) for 30 min at 37 °C. The fluorescent of ROS was measured using BD flow cytometry.

### 3.10. Quantification mRNA

U937 cells were seeded in 50 mm culture plates at a density of 1 × 10^6^ cells/well. The cells were treated under various conditions. After treatment, RNA was extracted from cells according to the instruction of RNA Isolation Kit, and the RNA was reverse transcribed to cDNA using Reverse Transcription Kit. The designed PCR primers were as follows: *Bcl-2* sense, 5′-CTGAGTACCTGAACCGGCA-3′ and antisense, 5′-GAGAAATCAAACA GAGGCCG-3′; *Bax* sense 5′-GGGTTGTCG CCCTTTTCTAC -3′ and antisense 5′-GGAGGAAGTCCAATGTCCAG-3′; *Bcl-X_L_* sense, 5′-TTCAGTGACCTGACATCCCA-3′ and antisense, 5′-CTGCTGCATTGTTCCCATAG -3′, and *β-actin* sense, 5′-CTGGAAGGTGGACAGCGAGG-3′ and antisense, 5′-GACGTGGACATCCGCAAAG-3′. The amplification system was performed using a SYBR Premix Ex Taq™ Kit. Real-time fluorescent quantitative Polymerase Chain Reaction (qRT-PCR) was performed using the ABI Prism 7500 Sequence Detection System (Foster City, CA, USA). 

### 3.11. Caspase-3, Caspase-9 Activity Assay

The activity of caspase-3 and caspase-9 were measured by Caspase-3 Activity Assay Kit ( and Caspase-9 Fluorescence Metric Assay Activity Assay Kit . U937 cells were seeded in 50 mm culture plates at a density of 5 × 10^6^ cells/well. The cells were treated under various conditions. After treatment, the cells were washed with cold PBS and centrifuged. The cells pellet was lysed, and the equal amounts of cell lysate were co-cultured with specific fluorogenic substrate. The activation of caspase-3 and caspase-9 was measured by fluorescence enzyme microplate reader.

### 3.12. Western Blotting Analysis

Caspase-9, caspase-3, and PARP were detected by Western blot method. The U937 cells were cultured at a density of 1 × 10^7^, and the cells were treated under various conditions. After treatment, the cells were washed with PBS, and the cell pellets were lysed in RIPA buffer containing 1 mM phenylmethanesulfonyl fluoride (PMSF). The same quantity of total proteins, measured using a bicinchoninic acid (BCA) Protein Assay Kit, were separated by 8–15% SDS-PAGE, then the proteins were transferred to polyvinylidene difluoride membranes (Burlington, MA, USA). The membranes were blocked with 5% non-fat dry milk in TBS-Tween20. Subsequently, membranes were incubated overnight at 4 °C with primary antibodies against caspase-3, caspase-9, PARP at 1:400 dilution, and washed three times with tris-buffered saline (TBS) and Tween-20 (TBST). Then, the membranes were incubated for 2 h with horseradish peroxidase-conjugated secondary antibodies at 1:1000 dilution and washed three times with TBST. The protein expression signals were visualized by Image Lab 4.0.1 software was from Bio-Rad (Hercules, CA, USA) after exposing the membranes to enhanced chemiluminescence solution, with the signals normalized to β-actin. 

### 3.13. Statistical Analysis

The results were represented as the means ± standard deviation (SD). Statistical significance was carried out by analysis of variance (ANOVA) test, then followed by Newman–Keuls multiple comparison test with GraphPad Prism version 5.00 for Windows (San Diego, CA, USA). *p* < 0.05 was considered statistically significant. These experiments were carried out with three independent experiments under the same conditions, and each experiment was performed with triplicate cultures.

## 4. Conclusions

In this paper, we provide a new strategy, by proposing BL irradiation to significantly enhance the proliferation inhibition of the leukemia cell U937, belonging to AML-M5 subtype, which has high efficiency compared treatment by the commonly used HHT drug. The molecular biology analysis indicates that the production of ROS and the decline of ΔΨm, as well as the varying genes of *Bcl-X_L_, Bcl-2,* and *Bax*, the activation of caspase-3 (caspase-9), and the cleavage of PARP, excited by BL irradiation, play a critical role in achieving high proliferation inhibition rate. Further considering the fewer side effects induced following treatment by BL irradiation, it is believed that this strategy could be helpful for improving the clinical treatment effects on various types of leukemia and tumors, as an alternative to some drugs and/or radiotherapy/chemotherapy that have some side effects.

## Supplementary Materials

The following are available online at http://www.mdpi.com/1422-0067/19/5/1464/s1.
