# Supplementary material for: Inhibition of Proliferation in U937 Cells Treated by Blue Light Irradiation and Combined Blue Light Irradiation/Drug
Source: Int J Mol Sci. 2018 May 15;19(5):1464. doi: 10.3390/ijms19051464 (PMC5983758; doi:10.3390/ijms19051464)
Supplement: Supplementary file 1 [file ijms-19-01464-s001.pdf]

International Journal of
Molecular Sciences

MDPI

Inhibition of Proliferation in U937 Cells Treated by Blue Light Irradiation and Combined Blue Light Irradiation/Drug

Jianjian Zhuang, Junsong Liu, Xuan Gao, Hongdong Li*

State Key Laboratory of Superhard Materials, Jilin University, Changchun 130012, China;
* Correspondence: hdli@jlu.edu.cn; Tel.: +86-431-85168881

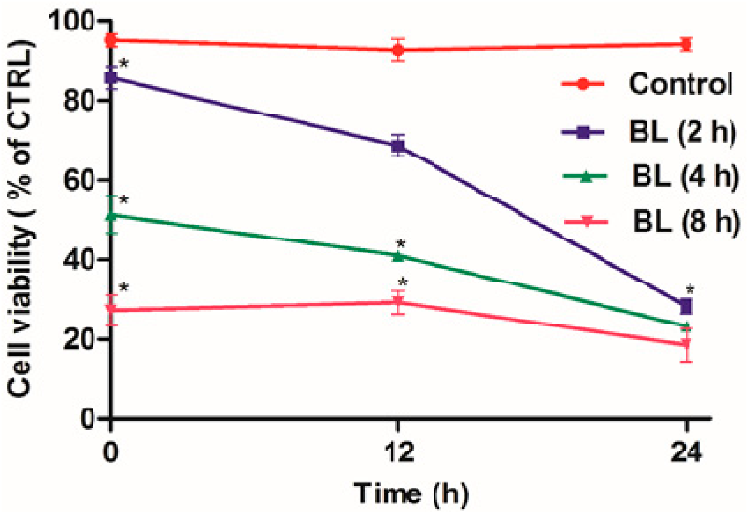

Figure S1. The proliferation inhibition of BL irradiation in U937 cells. The proliferation inhibition of U937 cells irradiated by BL for 2 h, 4 h, 8 h, and incubated for another 0 h, 12 h, 24 h. The cells viabilities are evaluated using cell CCK-8 assay, and the absorbance values are measured at 450 nm. These cells are divided into three groups for irradiation treatment, and the irradiation time is 2 h, 4 h and 8 h, respectively. Then, all cells are incubated respectively for 0 h, 12 h and 24 h. The proliferation inhibition ratios of cells irradiated for 2 h after being incubated for 0 h, 12 h and 24 h are 14.4%, 31.4%, 72.1%; cells irradiated for 4 h after being incubated for 0 h, 12 h and 24 h are 48.7%, 59.0%, 76.5%; cells irradiated for 8 h after being incubated for 0 h, 12 h and 24 h are 72.7%, 70.7%, 82.3%, respectively. Data shown are the means ± SD. * $p < 0.05$ vs control group.

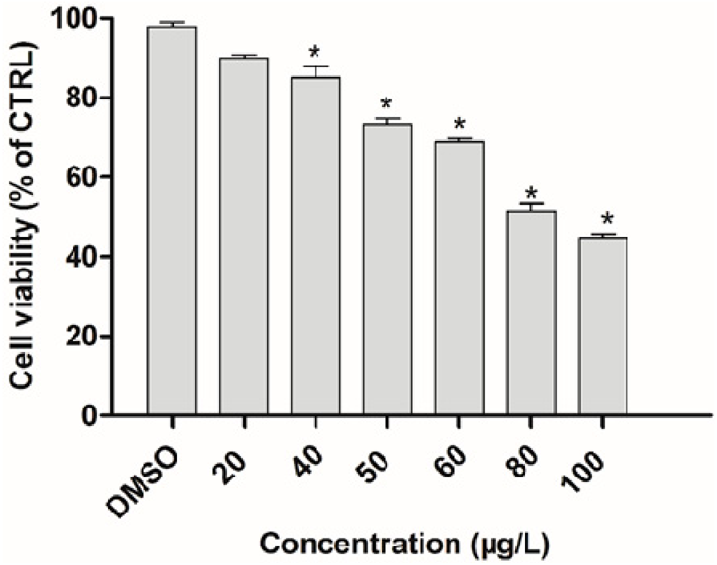

Figure S2. The proliferation inhibition of HHT treated U937 cells. The cells are incubated with the indicated concentration of HHT. After incubated for 24 h, the cells viabilities are evaluated using CCK-8 assay and the absorbance values are measured at 450 nm. Values shown are the means ± SD ($n = 3$). * $p < 0.05$ vs DMSO.

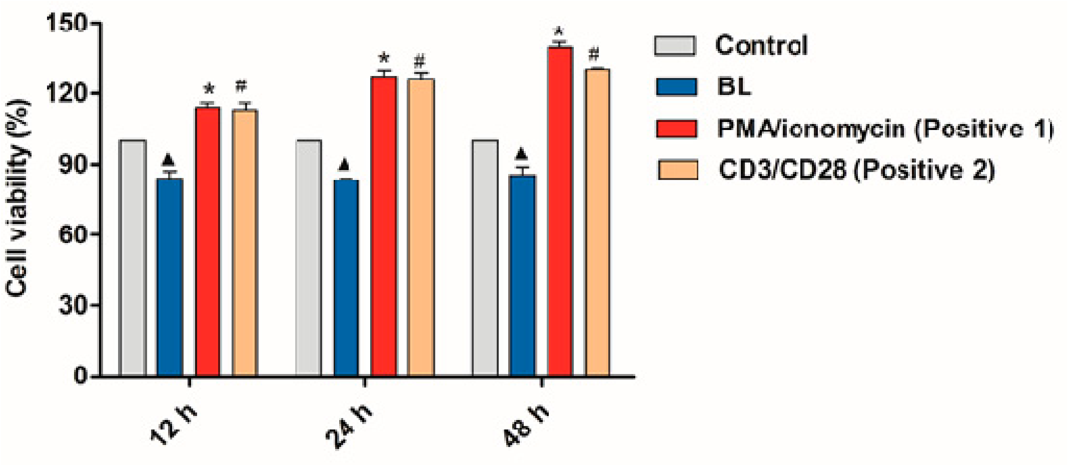

Figure S3. The specific proliferation inhibition of BL irradiation on PBMC. The isolated PBMC are irradiated by BL irradiation for 2 h, after incubated for 12 h, 24 h, 48 h. The cells viabilities are evaluated using CCK-8 assay, and the absorbance values are measured at 450 nm. PBMC cells are stimulated by PMA/ionomycin and CD3/CD28 antibody is set as Positive 1 and Positive 2, respectively. The control group is referred as 100% of viable cells. Values shown are the means ± SD ($n = 3$). ▲ $p < 0.05$ vs Control, * $p < 0.05$ vs Positive 1, # $p < 0.01$ vs Positive 2.
